# A Scoping Review: Financial Exploitation Among Older People Living With Dementia

**DOI:** 10.1093/geroni/igaf122.1363

**Published:** 2025-12-31

**Authors:** Wenxing Wei, Sarah Balser

**Affiliations:** Case Western Reserve University, Cleveland, Ohio, United States; Case Western Reserve University, Cleveland, Ohio, United States

## Abstract

Financial exploitation is among the most commonly reported forms of elder abuse, with older adults with dementia facing a heightened risk due to various vulnerabilities. However, limited research has specifically addressed this critical issue. Guided by the methodological framework of Arksey and O’Malley, this scoping review aims to summarize the existing literature to enhance current knowledge and raise awareness about financial exploitation in this vulnerable population. Following rigorous methods, we systematically searched four scientific databases (Ageline, Medline, CINAHL, and PsycINFO) for peer-reviewed articles published in English up to July 2024, identifying 21 articles for inclusion. A comprehensive data extraction process identified six key themes, including the prevalence of financial exploitation, the relationship between financial exploitation and cognitive function, risk factors and warning signs, case descriptions, preventive and intervention strategies, and barriers and facilitators to safeguarding. The findings emphasize the necessity for a multi-level safeguarding framework to prevent and address financial exploitation at the individual, professional, institutional, and systemic levels. This review also identifies critical gaps in existing literature, such as the need for research on protective factors, adverse effects, and barriers to implementing safeguarding measures. Research in this field is diverse yet fragmented, with limited studies directly addressing the intersection of financial exploitation and dementia. These insights underscore the need for future research, policy-making, and intervention development to better support and protect this at-risk population.

